# Supplementary figures and images for: Prediction of postoperative patient deterioration and unanticipated intensive care unit admission using perioperative factors
Source: PLoS One. 2023 Aug 3;18(8):e0286818. doi: 10.1371/journal.pone.0286818 (PMC10399824; doi:10.1371/journal.pone.0286818)

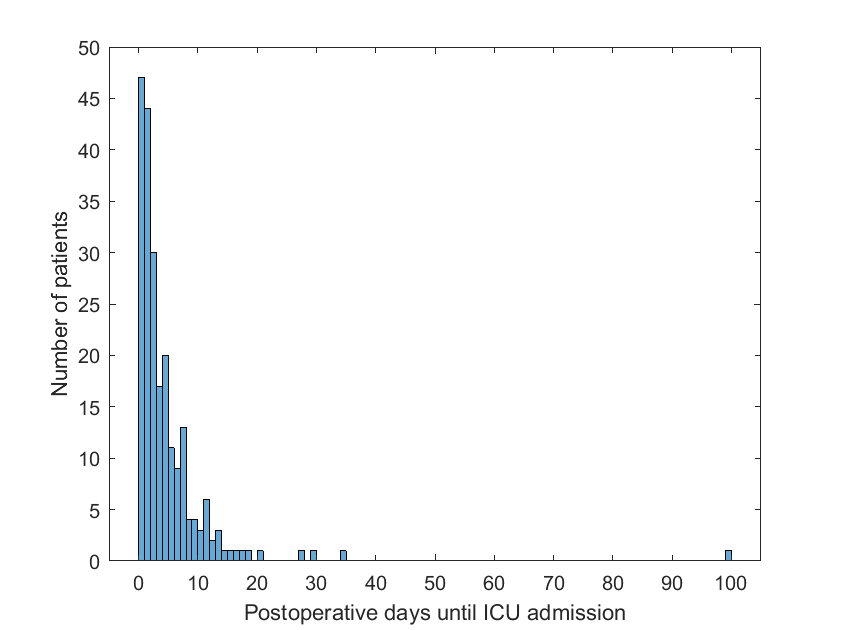

Supplement: S1 Fig — (TIF) [file pone.0286818.s001.tif]

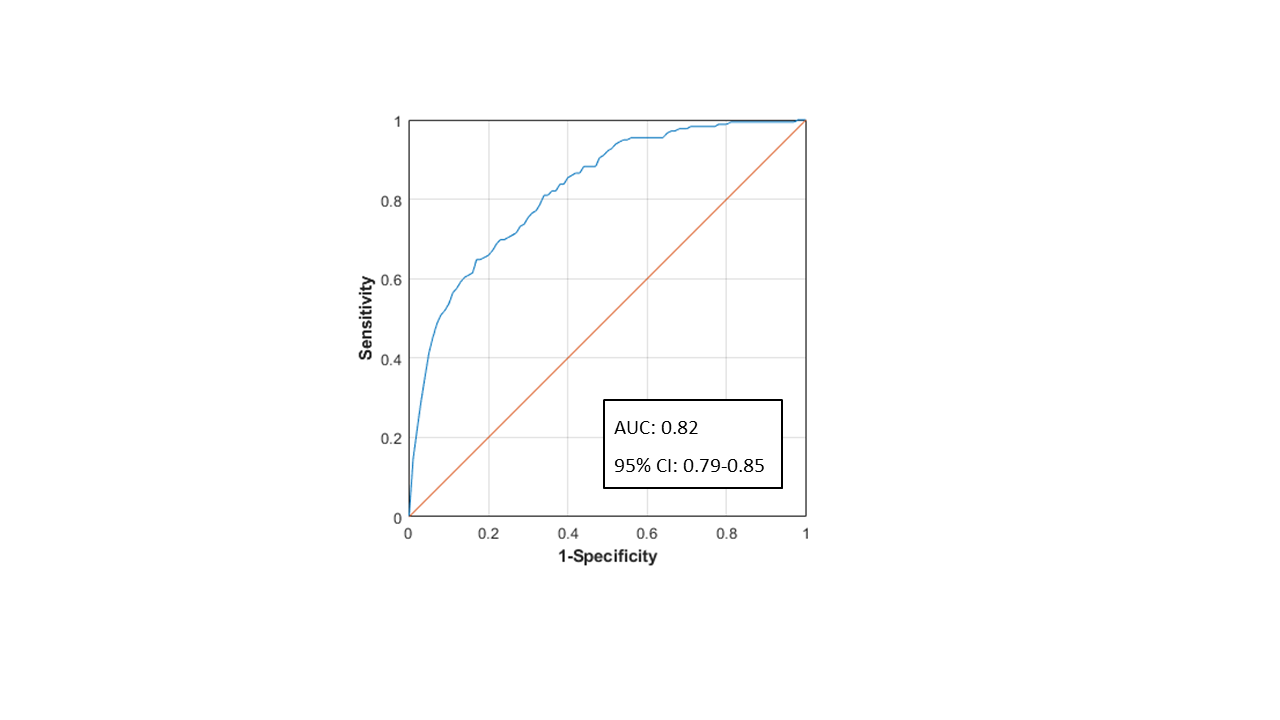

Supplement: S2 Fig — (TIF) [file pone.0286818.s002.tif]

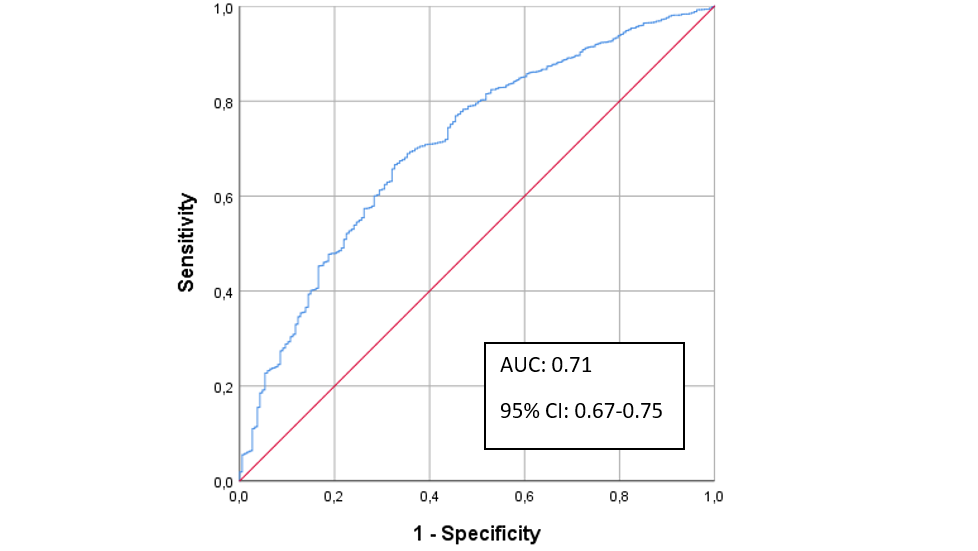

Supplement: S3 Fig — (TIF) [file pone.0286818.s003.tif]
