# Supplementary material for: Prediction of postoperative patient deterioration and unanticipated intensive care unit admission using perioperative factors
Source: PLoS One. 2023 Aug 3;18(8):e0286818. doi: 10.1371/journal.pone.0286818 (PMC10399824; doi:10.1371/journal.pone.0286818)
Supplement: S1 Table — (DOCX) [file pone.0286818.s004.docx]

**S1 Table. Collected variables.**

| Variable | Characteristics | Rationale |
| --- | --- | --- |
| Gender | Categorical | Gender is registered in the EMR for each patient. |
| Age, years | Continuous | Age is derived from date of birth and rounded to years. |
| BMI, kg/m^2^ | Continuous | BMI is calculated from last weight before surgery and height as registered in the EMR. |
| Specialism of surgery   - General surgery - Orthopedic surgery - Urological surgery - Gynecological surgery - Other | Categorical  Categorical  Categorical  Categorical  Categorical | Each category of specialism of surgery is registered in the EMR. |
| Acute surgery | Categorical | Label registered in the EMR |
| Method of anesthesia   - Narcosis - Narcosis/epidural - Spinal - Other | Categorical  Categorical  Categorical  Categorical | Labels registered in the EMR. |
| Comorbidities   - Diabetes mellitus - COPD/asthma - Hypertension - Alcohol abuse - History of smoking - Cerebrovascular accident - Heart failure - Chronic kidney failure - Cardiac arrhythmia - Thromboembolic event | Categorical  Categorical  Categorical  Categorical  Categorical  Categorical  Categorical  Categorical  Categorical  Categorical | Information on comorbidities is collected during preoperative screening by both patient and anesthesiologist. Either “yes” or “no” has to be answered to each comorbidity. If “yes” was answered, a field for explanation could be filled in. These fields were not used for analysis in this study. |
| ASA Physical Status Classification System score | Categorical | Labels registered in EMR by anesthesiologist. |
| Preoperative blood pressure, mmHg   - Systolic blood pressure - Diastolic blood pressure | Continuous  Continuous | Measured with oscillometric blood pressure cuff during preoperative screening by anesthesiologist. |
| Intraoperative signs of deterioration   - Heartrate >100 bpm - Minimum heartrate - Maximum heartrate - Oxygen saturation <90% - Oxygen saturation <85% - Continuous infusion of vasopressors - Continuous infusion of inotropes - Bolus administration of phenylephrine Number of boluses  Total dosage if used - Bolus administration of ephedrine Number of boluses  Total dosage - Administration of cell-salvaged blood - Red blood cell transfusion - Plasma transfusion - Platelet transfusion - Infusion of hydroxyethyl starch 6% | Categorical  Continuous  Continuous  Categorical  Categorical  Categorical  Categorical  Categorical  Continuous  Continuous  Categorical  Continuous  Continuous  Categorical  Categorical  Categorical  Categorical  Categorical | In the operating theatre, software is installed to collect data involving vital parameters, administered medication and anesthesiologists procedures such as placement of intravenous lines. This data is then stored in the EMR and available for extraction.  Heartrate: cut-off level of 100 bpm was chosen as this is most commonly used to calculate risk of deterioration and is more associated with pathophysiological conditions. Minimum and maximum heart rate were included for analysis to evaluate whether such variables are associated to deterioration.  It was decided to assess blood pressure or circulation by means of vasopressor use, since it was assumed that vasopressors are indicated when the anesthesiologist judges that the circulation is impaired. |
| Surgery duration, minutes | Continuous | Transfer times are logged in operating theatre. Surgery duration was defined as the time in minutes between first incision and last suture by the surgeon. These timestamps are known to be reliably recorded. |
| Time in operating theatre, minutes | Continuous | Transfer times are logged in the operating theatre, however these timestamps are prone to administrative flaws. Time in operating theatre was defined as time between first registered heart rate and last registered heart rate in operating theatre as these are automatically and more precisely recorded. |
| Postoperative data   - Heartrate >100 bpm - Minimum heartrate - Maximum heartrate - Oxygen saturation <90% - Oxygen saturation <85% - Red blood cell transfusion - Infusion of hydroxyethyl starch 6% - Anesthesiologists’ review required Number of reviews - Duration of PACU stay, minutes | Categorical  Continuous  Continuous  Categorical  Categorical  Categorical  Categorical  Categorical  Continuous  Continuous | The postoperative data consists of data from the recovery room. The same rationale as for the peroperative data applies, except for anesthesiologists’ review. Information from anesthesiologists’ review was manually entered in the software in OR and extracted for this study to assess whether it could be associated with postoperative unanticipated ICU admission.  Duration of PACU stay was defined as the time between first and last registered heart rate in PACU. Rationale for this definition is explained for time in OR. |

*ASA: American Society Anesthesiologists; EMR: Electronic Medical Record; PACU: Post Anesthesia Care Unit.*
