# Supplementary material for: Prediction of postoperative patient deterioration and unanticipated intensive care unit admission using perioperative factors
Source: PLoS One. 2023 Aug 3;18(8):e0286818. doi: 10.1371/journal.pone.0286818 (PMC10399824; doi:10.1371/journal.pone.0286818)
Supplement: S2 Table — (DOCX) [file pone.0286818.s005.docx]

**S2 Table. Baseline characteristics and outcomes.**

|  | Cases | Controls | p-value |
| --- | --- | --- | --- |
| Total number of patients | 223 | 25296 | NA |
| Male gender, number (%) | 109 (48.9) | 10002 (39.5) | 0.005 |
| Age, median years (IQR) | 67 (19) | 55 (27) | <0.001 |
| BMI, median (IQR) | 26.6 (8.4) | 27.1(9.2) | 0.048 |
| Length of stay, median days (IQR) |  |  |  |
| Hospital | 15.0 (13.9) | 1.3 (2.3) | <0.001 |
| ICU | 1.7 (3.2) | NA | NA |
| Mortality, number (%) |  |  |  |
| Hospital | 31 (13.9) | 43 (0.2) | <0.001 |
| 28-day | 4 (1.8) | 80 (0.3) | 0.006 |
| 90-day | 7 (3.1) | 180 (0.7) | 0.001 |
| Type of surgery, number (%) |  |  |  |
| General | 164 (73.5) | 12.987 (51.3) | <0.001 |
| Orthopedic | 24 (10.8) | 3788 (15.0) | 0.079 |
| Urological | 13 (5.8) | 2775 (11.0) | 0.014 |
| Gynecological | 6 (2.7) | 2759 (10.9) | <0.001 |
| Other | 16 (7.2) | 2987 (11.8) | 0.033 |
| Acute surgery, number (%) | 37 (16.6) | 2879 (11.4) | 0.015 |
| Method of anesthesia, number (%) |  |  |  |
| General | 149 (66.8) | 19.433 (76.8) | <0.001 |
| General and epidural | 59 (26.5) | 1080 (4.3) | <0.001 |
| Spinal | 12 (5.4) | 3726 (14.7) | <0.001 |
| Other | 3 (1.3) | 1057 (4.2) | 0.035 |
| Comorbidities, number (%) |  |  |  |
| Diabetes mellitus | 44 (23.4) | 2466 (11.1) | <0.001 |
| COPD/asthma | 27 (14.4) | 2749 (12.4) | 0.410 |
| Hypertension | 74 (39.9) | 8950 (26.8) | <0.001 |
| Alcohol abuse | 12 (6.4) | 1197 (5.4) | 0.548 |
| History of smoking | 43 (22.9) | 4787 (21.6) | 0.678 |
| Cerebrovascular accident | 14 (7.4) | 888 (4.0) | 0.017 |
| Heart failure | 37 (19.4) | 3203 (14.4) | 0.050 |
| Chronic kidney failure | 10 (5.3) | 848 (3.8) | 0.285 |
| Cardiac arrhythmia | 28 (14.9) | 2834 (12.8) | 0.382 |
| Thromboembolic event | 15 (8.1) | 987 (4.5) | 0.018 |
| Use of vitamin K antagonists | 37 (16.6) | 1024 (4.0) | <0.001 |
| Use of direct oral anticoagulants | 10 (4.5) | 1996 (7.9) | 0.060 |
| Use of antiplatelet drugs* | 32 (14.3) | 834 (3.3) | <0.001 |
| ASA score, median (IQR) | 2 (1) | 2 (0) | <0.001 |
| Missing | 19 | 2380 | NA |
| Preoperative data, median (IQR) |  |  |  |
| Systolic blood pressure mmHg | 132.0 (24.75) | 130 (25) | 0.780 |
| Diastolic blood pressure mmHg | 75 (16.75) | 80 (15) | 0.001 |
| Peroperative signs of deterioration, number (%) |  |  |  |
| Heart rate >100 bpm | 106 (47.5) | 9686 (38.9) | 0.005 |
| Minimum heart rate | 55 (17) | 54 (13) | 0.019 |
| Maximum heart rate | 100 (24) | 95 (25) | 0.001 |
| Oxygen saturation <90% | 74 (33.2) | 6920 (27.4) | 0.052 |
| Continuous infusion of vasopressors** | 24 (10.8) | 1198 (4.7) | <0.001 |
| Continuous infusion of inotropes*** | 1 (0.4) | 5 (0.0) | 0.051 |
| Bolus administration of phenylephrine | 112 (50.2) | 4506 (17.8) | <0.001 |
| Number of boluses if used, median (IQR) | 3 (3.75) | 3 (3) | 0.023 |
| Total dosage if used, median (IQR) | 400 (594) | 350 (400) | 0.001 |
| Bolus administration of ephedrine | 131 (58.7) | 11.953 (47.3) | 0.001 |
| Number of boluses if used, median (IQR) | 2 (2) | 2 (2) | 0.019 |
| Total dosage if used, median (IQR) | 15 (12.5) | 12.5 (12.5) | 0.021 |
| Administration of tranexamic acid | 16 (7.2) | 494 (2.0) | <0.001 |
| Administration of cell-salvaged blood | 2 (0.9) | 257 (1.0) | 0.860 |
| Red blood cell transfusion | 11 (4.9) | 128 (0.5) | <0.001 |
| Plasma transfusion | 2 (0.9) | 14 (0.1) | 0.008 |
| Platelet transfusion | 2 (0.9) | 7 (0.0) | 0.003 |
| Infusion of hydroxyethyl starch 6% | 26 (11.7) | 638 (2.5) | <0.001 |
| Surgery duration, median in minutes (IQR) | 89 (68.0) | 52 (48) | <0.001 |
| Time in operating room, median in minutes (IQR) | 126 (89.5) | 77 (59) | <0.001 |
| Postoperative data, number (%) |  |  |  |
| Heart rate >100 bpm | 96 (43) | 5883 (23.3) | <0.001 |
| Minimum heart rate, median (IQR) | 70 (18) | 63 (18) | <0.001 |
| Maximum heart rate, median (IQR) | 97 (23) | 88 (23) | <0.001 |
| Oxygen saturation <90% | 120 (53.8) | 9570 (37.8) | <0.001 |
| Oxygen saturation <85% | 61 (27.4) | 3342 (13.2) | <0.001 |
| Red blood cell transfusion | 6 (2.7) | 67 (0.3) | <0.001 |
| Plasma transfusion | 0 (0.0) | 5 (0.0) | <0.001 |
| Platelet transfusion | 0 (0.0) | 4 (0.0) | 1.000 |
| Infusion of hydroxyethyl starch 6% | 7 (3.1) | 109 (0.4) | 1.000 |
| Anesthesiologist review required | 29 (13.0) | 1295 (5.1) | <0.001 |
| Duration of PACU stay in minutes, median (IQR) | 102 (70) | 74 (37) | <0.001 |

*dipyridamole, clopidogrel, ticagrelor **norepinephrine or phenylephrine, ***dobutamine or milrinone
