# Supplementary material for: Prediction of postoperative patient deterioration and unanticipated intensive care unit admission using perioperative factors
Source: PLoS One. 2023 Aug 3;18(8):e0286818. doi: 10.1371/journal.pone.0286818 (PMC10399824; doi:10.1371/journal.pone.0286818)
Supplement: S3 Table — (DOCX) [file pone.0286818.s006.docx]

**S3 Table. Results of univariate analysis.**

|  | OR | CI (95%) | p-value |
| --- | --- | --- | --- |
|  |  |  |  |
| Male | 1.46 | 1.12-1.90 | 0.005 |
| Age, years | 1.03 | 1.02-1.04 | <0.001 |
| BMI, kg/m^2^ | 0.98 | 0.96-1.00 | 0.36 |
| Specialism of surgery   - General surgery - Orthopedic surgery - Urological surgery - Gynecological surgery - Other | 2.635  0.685  0.502  0.226  0.577 | 1.955-3.551  0.448-1.047  0.287-0.881  0.100-0.509  0.347-0.961 | <0.001  0.081  0.016  <0.001  0.035 |
| Acute surgery | 1.55 | 1.09-2.21 | 0.016 |
| Anesthesia technique   - General - General and epidural - Spinal - Other | 0.607  8.067  0.329  0.313 | 0.459-0.804  5.954-10.929  0.184-0.590  0.100-0.979 | <0.001  <0.001  <0.001  0.046 |
| Comorbidities   - Diabetes mellitus - COPD/asthma - Hypertension - Alcohol abuse - History of smoking - Cerebrovascular accident - Heart failure - Chronic kidney failure - Cardiac arrhythmia - Thromboembolic event | 2.45  1.19  1.82  1.20  1.08  1.93  1.43  1.42  1.20  1.88 | 1.74-3.44  0.79-1.79  1.35-2.43  0.67-2.16  0.76-1.51  1.12-3.35  1.00-2.05  0.75-2.69  0.80-1.79  1.11-3.20 | <0.001  0.41  <0.001  0.55  0.68  0.02  0.05  0.29  0.38  0.02 |
| ASA Physical Status Classification System score | 2.62 | 2.12-3.23 | <0.001 |
| Preoperative blood pressure, mmHg   - Systolic blood pressure - Diastolic blood pressure | 1.00  0.98 | 0.99-1.01  0.97-0.99 | 0.62  0.00 |
| Intraoperative signs of deterioration   - Heart rate >100 bpm - Lowest heart rate - Highest heart rate - Oxygen saturation <90% - Oxygen saturation <85% - Administration of vasopressors - Administration of inotropes - Bolus administration of phenylephrine Number of boluses  Total dosage if used - Bolus administration of ephedrine Number of boluses  Total dosage - Transfusion of cell-salvaged blood - Transfusion of red blood cells - Transfusion of plasma - Transfusion of thrombocytes - Infusion of hydroxyethyl starch 6% | 1.46  1.02  1.01  1.32  1.03  2.43  22.79  4.66  1.27  1.00  1.59  1.21  1.07  0.88  10.20  16.34  32.69  5.10 | 1.12-1.9065  1.00-1.03  1.00-1.02  1.00-1.75  0.70-1.53  1.58-3.72  2.65-195.82  3.57-6.07  1.22-1.31  1.00-1.00  1.22-2.08  1.12-1.30  1.02-1.04  0.22-3.57  5.43-19.17  3.69-72.33  6.75-158.26  3.36-7.74 | 0.01  0.01  0.00  0.05  0.88  <0.001  0.004  <0.001  <0.001  <0.001  0.001  <0.001  <0.001  0.86  <0.001  <0.001  <0.001  <0.001 |
| Surgery duration, minutes | 1.001 | 1.01-1.01 | <0.001 |
| Time in operating theatre, minutes | 1.01 | 1.01-1.01 | <0.001 |
| Postoperative data   - Heart rate >100 bpm - Lowest heart rate - Highest heart rate - Oxygen saturation <90% - Oxygen saturation <85% - Red blood cell transfusion - Infusion of hydroxyethyl starch 6% - Anesthesiologists’ review required Number of reviews - Time in PACU, minutes | 2.26 1.04  1.02  1.91  2.47  10.41  7.49  2.77 2.14  1.02 | 1.73-2.96 1.03-1.05  1.02-1.03  1.47-2.49  1.84-3.33  4.47-24.26  3.45-16.27  1.87-4.11  1.70-2.70  1.01-1.02 | <0.001 <0.001  <0.001  <0.001  <0.001  <0.001  <0.001  <0.001  <0.001  <0.001 |

*ASA: American Society Anesthesiologists; BMI: Body Mass Index; PACU: Post Anesthesia Care Unit.*
