# Supplementary material for: Prediction of postoperative patient deterioration and unanticipated intensive care unit admission using perioperative factors
Source: PLoS One. 2023 Aug 3;18(8):e0286818. doi: 10.1371/journal.pone.0286818 (PMC10399824; doi:10.1371/journal.pone.0286818)
Supplement: S4 Table — (DOCX) [file pone.0286818.s007.docx]

**S4 Table. Predictors after bootstrapping.**

| BNames | OR_MEAN | OR_5 | OR_95 |
| --- | --- | --- | --- |
| Intercept | 0,004365119 | 0,003653859 | 0,005181838 |
| DURATION OF pacu STAY | 1,372072408 | 1,254563579 | 1,478833224 |
| Narcosis | 1 | 1 | 1 |
| Narcosis + ePIDURAL | 1,170853325 | 1,088128446 | 1,26000197 |
| Narcosis oTHER | 0,9010618 | 0,788282553 | 0,986761283 |
| Narcosis sPINAL | 0,967596104 | 0,865751329 | 1 |
| bOLUS ADMINISTRATION OF PHENYLEPHRINE IN or | 1,178592906 | 1,079450354 | 1,283003343 |
| tIME IN OPERATING THEATRE | 1,201802346 | 1,005104689 | 1,337577072 |
| sURGERY dURATION | 1,047334281 | 1 | 1,215355027 |
| ASA 1 | 0,856878963 | 0,730234819 | 0,998607818 |
| ASa 2 | 1 | 1 | 1 |
| ASA 3 | 1,228441437 | 1,124681144 | 1,347378815 |
| ASA 4 | 1,002693481 | 0,978802622 | 1,027389148 |
| INFUSION OF HYDROXYETHYL STARCH dURING or | 1,007449997 | 0,970504165 | 1,052976733 |
| mINIMUM hEART RATE IN pacu | 1,198502436 | 1,060742437 | 1,330201085 |
| Age | 1,178376219 | 1,037964034 | 1,339458735 |
| rED BLOOD CEL TRANSFUSION DURING SURGERY | 1,047440763 | 1,001273023 | 1,087611356 |
| mAXIMUM HEART RATE IN pacu | 1,042235801 | 1 | 1,145706968 |
| SATURATION BELOW 85% IN pacu | 1,073294373 | 1 | 1,173910857 |
| HEART RATE ABOVE 100 IN pacu | 1,095678403 | 1 | 1,240816588 |
| rED BLOOD CELL TRANSFUSION IN pacu | 1,015115137 | 0,990924245 | 1,050742042 |
| Diabetes | 1,074263255 | 1 | 1,17383987 |
| iNFUSION OF HYDROXYETHYL STARCH DURING or | 0,98504562 | 0,940115443 | 1,010477574 |
| aNESTHESIOLOGISTS’ REVIEW REQUIRED | 0,986355022 | 0,925524683 | 1 |
| sATURATION BELOW 90% IN pacu | 0,999031401 | 0,994501861 | 1,015940192 |
| pLATELET TRANSFUSION IN or | 1,037555757 | 1 | 1,064556064 |
| sPECIALISM: gENERAL SURGERY | 1,57283319 | 1,346583291 | 1,828807018 |
| sPECIALISM: gYNECOLOGICAL SURGERY | 0,994705522 | 0,959214326 | 1 |
| sPECIALISM: oRTHOPEDIC sURGERY | 0,9784797 | 0,897990956 | 1 |
| sPECIALISM:  oTHER | 0,998447007 | 0,990352246 | 1 |
| sPECIALISM: uROLOGICAL SURGERY | 0,998832683 | 1 | 1 |
| cONTINUOUS INFUSION OF VASOPRESSORS | 0,967622046 | 0,885624999 | 1 |
| hYPERTENSION | 1,058396181 | 1 | 1,147918966 |
| pLASMA TRANSFUSION IN or | 1,020441429 | 0,974480062 | 1,056127971 |
| bOLUS ADMINISTRATION OF EPHEDRINE | 0,982245411 | 0,903075152 | 1 |
| pREOPERATIVE dIASTOLIC BLOOD PRESSURE | 0,929278931 | 0,843412139 | 1 |
| mAXIMUM HEART RATE IN or | 0,993724708 | 0,951890027 | 1 |
| Sex | 1,009641959 | 1 | 1,061877801 |
| hEART RATE ABOVE 100 IN or | 1,003009523 | 0,978885336 | 1,0488498 |
| mINIMUM HEART RATE IN or | 1,065913105 | 1 | 1,159739822 |
| aCUTE SURGERY | 1,061468061 | 1 | 1,163433016 |
| cEREBROVASCULAR ACCIDENT | 1,00557641 | 0,978332781 | 1,046686174 |
| THROMBOEMBOLIC EVENT | 1,045446269 | 1 | 1,133300274 |
| BMI | 0,900870691 | 0,801106181 | 1 |
