# Supplementary material for: Prediction of postoperative patient deterioration and unanticipated intensive care unit admission using perioperative factors
Source: PLoS One. 2023 Aug 3;18(8):e0286818. doi: 10.1371/journal.pone.0286818 (PMC10399824; doi:10.1371/journal.pone.0286818)
Supplement: S5 Table — (DOCX) [file pone.0286818.s008.docx]

**S5 Table. Types of organ dysfunction as underlying reason for unanticipated ICU admission.**

| Type of organ dysfunction | Number of cases (%) | Specific disorders |
| --- | --- | --- |
| Cardiovascular | 110 (49.3) | Sepsis and septic shock  SIRS/hypotension Hypertension Cardiac decompensation (Acute) coronary event In-hospital cardiac arrest |
| Hematological | 51 (22.9) | Major bleeding Pulmonary embolism Cerebral infarction |
| Respiratory | 44 (19.7) | PneumoniaAspiration (combined with vocal cord lesion) Pleural fluid Hypoventilation due to abdominal cause Undiagnosed OSAS Interstitial pneumonitis |
| Central nervous system | 12 (5.4) | (Relative) opioid intoxication Vasoplegia due to epidural anesthesia Monitoring during severe pain management |
| Metabolic | 5 (2.2) | Hypokalemia  Symptomatic hyponatremia Alkalosis |
| Renal | 1 (0.4) | Hyperkaliemia |

*OSAS: obstructive sleep apnea syndrome; SIRS: systemic inflammatory response syndrome.
An infectious cause was determined based on a combination of clinical parameters, increased inflammatory lab results and sometimes positive cultures for which antibiotics were started.*
