# Supplementary material for: Prediction of postoperative patient deterioration and unanticipated intensive care unit admission using perioperative factors
Source: PLoS One. 2023 Aug 3;18(8):e0286818. doi: 10.1371/journal.pone.0286818 (PMC10399824; doi:10.1371/journal.pone.0286818)
Supplement: S6 Table — Each category is further subdivided into involved organ systems as underlying reason for unanticipated ICU admission. (DOCX) [file pone.0286818.s009.docx]

**S6 Table. Interventions in ICU during unanticipated ICU admission. Each category is further subdivided into involved organ systems as underlying reason for unanticipated ICU admission.**

| Intervention | Number of cases | % of total unanticipated ICU admissions |
| --- | --- | --- |
| Antibiotics | 156 | 70.0 |
| Cardiovascular | 93 | 41.7 |
| Hematological | 18 | 8.1 |
| Respiratory | 40 | 17.9 |
| Central nervous system | 3 | 1.3 |
| Metabolic | 2 | 0.9 |
| Vasopressors | 87 | 39.0 |
| Cardiovascular | 54 | 24.2 |
| Hematological | 15 | 6.7 |
| Respiratory | 16 | 7.2 |
| Central nervous system | 1 | 0.4 |
| Metabolic | 1 | 0.4 |
| Inotropes | 21 | 9.4 |
| Cardiovascular | 13 | 5.8 |
| Hematological | 4 | 1.8 |
| Respiratory | 4 | 1.8 |
| Mechanical ventilation | 49 | 22.0 |
| Cardiovascular | 28 | 12.6 |
| Hematological | 6 | 2.7 |
| Respiratory | 14 | 6.3 |
| Metabolic | 1 | 0.4 |
