# Supplementary material for: Prediction of postoperative patient deterioration and unanticipated intensive care unit admission using perioperative factors
Source: PLoS One. 2023 Aug 3;18(8):e0286818. doi: 10.1371/journal.pone.0286818 (PMC10399824; doi:10.1371/journal.pone.0286818)
Supplement: Attachment — Submitted filename: Response to reviewers.docx [file pone.0286818.s010.docx]

**Response to reviewers**

Reviewer #1: Overall comments:
This study was based in a large tertiary hospital in The Netherlands and sought to develop a prediction model utilising pre, intra- and post-operative factors to predict an unanticipated ICU admission. An unanticipated ICU admission was defined as the patient being discharged from PACU to the ward, but then needing ICU admission. Over 25,000 patients were included, with 223 patients meeting the definition of a case. Using factors from the pre, intra- and post-operative the AUROC was highest, compared to only included pre-operative or pre- and intra-operative factors.

I have structured my reviews below with section and line specific comments. But firstly some over-arching comments.

I would recommend major revisions for this paper. In the current form it is not appropriate for publication. However, the paper is well written, methodology relatively sound and conclusions not far-reaching. If the below suggestions can be considered and if possible addressed I would be very happy to re-review this paper and consider it for acceptance.

*Author response: Thank you for the thorough review of our manuscript. We appreciate the opportunity to improve our manuscript with your valuable suggestions. Our response to each of the points can be found bellow including improvements made to the manuscript text. We have supplied a modified version of the manuscript using track changes, page and line numbers below refer to place the changes were made in the location in the manuscript with tracked changes.*

There are 4 main problems that must be addressed:
#1 - is this paper seeking to create a prediction tool or compare the type of data (pre/intra/post-operative data) included in the tool to determine which is best? I would suggest including all data that will be known at the time of making the decision. If the authors disagree that is fine, but it needs to be clear in all sections of the paper, the abstract and methods state you are comparing how different data influences the model, but the introduction does not. Further, I am not clear on the benefit of assessing what data types to include, why not include all available data and build the best possible model? It sounds like all data is available in the EMR anyway?

*Author response: Thank you for pointing out that the goal of this paper could be more clearly presented. The first option of the reviewer, a prediction or risk tool, is exactly the reason this study was performed and this paper was written. It might be good to explain the context more elaborately:*

*The type of EMR changed recently in 2018 and is now more advanced to collect and extract data and possibly to implement prediction or risk tools. The data from the old EMR was investigated to see which variables to include. The old EMR was chosen to collect data since we expected that a large amount of data was needed for the rare outcome of unanticipated ICU admissions.*

*In addition, in our hospital, patients with anticipated ICU admission in preoperative assessment are transferred directly to the ICU. The other group of patients go to PACU first. At the end of their PACU stay, the anesthesiologist decides to transfer the patient to the general ward or to the ICU. Therefore the time of decision making is at the end of PACU stay. And therefore, all data that could be known at the time of decision making is included.*

*The reviewer made us realize that the addition of the different data combinations is confusing. Indeed, to answer the research question and hypothesis, the only analysis needed is to build the best possible model by including all available data for analysis. We were merely establishing a baseline by showing the models with only preoperative and pre- and intraoperative and show the reader the influence of adding PACU data.*

*To improve the manuscript to explain the above mentioned suggestions, it was more clearly pointed out in the Introduction that the future goal is an automatically calculated risk score by adding the following sentence in Line 73: “With the implementation of more advanced EMRs, the readily available data from all perioperative stages in the EMR could improve the development of clinical decision support tools in the PACU by assigning patients an automatically calculated risk score for unanticipated ICU admission.”*

*To point out more clearly that the data was analyzed for clinical decision support in PACU, the words “in post anesthesia care units” was added in Line 95.*

*The following sentence was removed from Line 88 to avoid double information: “In addition, the readily available data from all perioperative stages in the EMR could improve the development of clinical decision support tools in the PACU.”*

*Regarding what data types to include, we agree that it is best to use the model with all available data. We removed the other models from the main manuscript and placed them in the supplementary information section as S1 Figure and S2 Figure.*

#2 - Data is included in the model that would not be available when you wish to use the model. Looking through table 1 the 4 PACU period variables would not be known at the time of using the model (pre PACU discharge). The authors need to redo their analysis with only variables known prior to PACU discharge

*Author response: The reviewer makes clear that it was insufficiently explained how work flow and the eventual tool would make use of the data in Table 1. The anesthesiologist decides at the end of PACU stay which location the patient will be transferred to. At that moment, there is information on the use of vasopressors, already spent time in PACU etcetera that the digital tool in the EMR could use as input to calculate a risk score. Therefore, all data that is needed is available at the moment the prediction is made.*

*We take the point that is was not so clear in the original submission. A figure was included to visualize how our hospital flow works and which data can be included during the different periods. This figure could also show where such a tool might be used.*

*This figure (Fig 1) was added to the Methods section in Line 123 as text and in Line 126 as Figure. The other Figure numbers were adjusted where appropriate.*

#3 - Is there an upper limit for how long post PACU discharge an ICU admission was defined as a case? If you have included ICU admissions >2-3 days after being on the ward I worry if you case cohort is not homogenous. The reasons and contributing factors to ICU admission immediately after PACU discharge and after being on the ward for >2-3 days are very different. I would advocate for you to report median/mean + IQR/SD (or better yet a histogram) for the time interval from PACU discharge to ICU admission to help readers understand and limit your cohort to not include admissions after >2-3 days post PACU discharge.

*Author response: The median time between PACU discharge and unanticipated ICU admission was 2.68 days (IQR 4.61 days). Therefore, the case group consists of mostly early unanticipated ICU admission, which is in line with the inclusion criteria in a comparable study by Petersen Tym (reference 5 in the manuscript). Since this complication is so rare, it seems reasonable to include all unanticipated ICU admission as long as they were happening during the same postoperative stay in the hospital. The reviewer points out correctly that the reason and factors for early versus late unanticipated ICU admission are heterogeneous, but regardless of the limit in post PACU stay the group will be heterogeneous. Although it might seem logical that ICU admissions <2-3 days after surgery are more related to perioperative factors than ICU admission >2-3 days, we did not want to exclude this group beforehand to explore if perioperative data could predict later ICU admission as well.*

*We would like to follow the reviewer’s idea of including a histogram to discuss this in the limitations section.*

*The following sentences were added to Results section in Line 188: “The median time between PACU discharge and unanticipated ICU admission was 2.68 days (IQR 4.61 days). A histogram on these time spans is presented in S1 Figure.”*

#4 - You need to explain how this model can be used practically. Would it be implemented into an EMR? Or can you choose 4-5 of the variables, assign scores for each variable that contribute to a global score that corresponds with a X% change of needing to go to ICU unexpectedly. Without clearly providing a path for implementation a model is of little benefit to the medical world and most importantly, patients. Practically speaking if you could create a tool for readers that uses your data to assign a % chance of having an unplanned ICU admission that would be helpful. The tools I use in my day to day practice as easy to calculate, contain few variables and give me a low / medium / high risk fo X outcome.

*Author response: We are happy to read the reviewer is as enthusiastic about the possible practical use of the model as we are. The intended use of this model would be to implement it in into the EMR and automatically assign a patient who reaches the end of his PACU stay a low / medium/ high risk for unanticipated ICU admission later on. The reason we did not report on this tool is because we did not do the analysis yet and have not yet validated the tool.*

*As mentioned before in the response to main problem #1 on page 2 of this document, we adjusted the manuscript to increase clarity on the intended use of this study: a digital tool in the new EMR that automatically calculates a risk score for unanticipated ICU admission.*

Abstract:
L41 Post anaesthesia care unit stay - add "length of" to the start. Currently it sounds like a binary variable.

*Author response: ‘length of” was added.*

Introduction:
- Overall a well written introduction. Authors often struggle to explain the "why" for prediction tools like this, but you have done this well. One overall comment is the final section of the introduction leads me to believe you are aiming to create a prediction tool, whereas your abstract makes it sounds like you are assessing the benefit of including intra- and postoperation factors to a tool that already has pre-operative factors (particularly in your abstract conclusion). As a reader I am left wondering - are these authors creating a new tool? Or are they trying to alter an existing tool to make it better? You need to clarify this so readers don't get confused and have a clear path of reading to follow.

*Author response: For the answer to this question, I would like to refer to our response to main problem #1 and #4 in this document.*

L59 The authors reference critical care admissions -> I would stick with referring to ICU admissions for consistency.

*Author response: Thank you for pointing out this inconsistency, this was changed accordingly. .*

L66 This line needs rewording "Most clinical experience..." -> I would suggest changing it to "Clinical experience and knowledge are primarily used to support clinical decision making..."

*Author response: Thank you for the suggestion. The sentence was changed according to the reviewer’s suggestion in Line 67.*

L68 would suggest changing brain overload to cognitive overload

*Author response: The wording was changed in Line 69.*

Methods:
- Overall a clear methods section. A few things need to be clarified, primarily the method of manual case review. Further, you need to clarify if there is an upper bound for how long post PACU discharge an ICU admission is considered a case. If you are including patients that go to ICU >2-3 days post PACU discharge this may impact your models ability to predict these admissions. As one can imagine intra and post-operative factors are unlikely to predict ICU admission 2-3 days after the surgery.

*Author response: to answer this valuable reviewer’s remark, I would like to refer to our response to main problem #3 on page 3 of this document.*

L94 Unsure if this is specific to regulations in The Netherlands (if so please disregard my suggestion), but including the date and chairperson of the committee that approved your project is probably unnecessary. The ethics committee and reference number is sufficient.

*Author response: This information is not required indeed, therefore the superfluous information was removed from Line 102.*

L96 STROBE guidelines are not appropriate here. You need to use the TRIPOD guidelines. (<https://www.equator-network.org/reporting-guidelines/tripod-statement/>)

Author response: The STROBE statement was replaced by the TRIPOD according to the reviewer’s suggestion.

L97 "In this hospital" - I suggest rewording this. Perhaps "The study hospital performs approximately 7400 surgical procedures and admits 3000 patients to ICU annually"

*Author response: This suggestion was adjusted in the manuscript in Line 104-106.*

L100 Suggest changing "large abdominal surgery" to "major abdominal surgery"

*Author response: This suggestion was adjusted in the manuscript in Line 108.*

L101 Could you explain the psychiatric patients, are these post overdose? If so I would suggest reporting as "drug overdose" instead. When I hear "psychiatric patients" I immediately think they are transfers from a psychiatric unit.

*Author response: Indeed, drug overdose is more appropriate, this term will replace psychiatric patients in Line 109.*

L115 I would suggest adding a line to state you did a manual review of unanticipated ICU admission cases identified by your HR rule. You describe this process in Figure 1, and mention it in the results, but this is not 100% clear. Something like "The authors manually reviewed all 285 cases identified by the HR rule and excluded any instances where an unanticipated ICU admission did not occur, or if appropriate moved them to the control group"

*Author response: Thank you for noticing the missing description in the Methods section and proposing an adequate sentence. The proposed sentence was included in the manuscript in Line 127-129.*

Results:
- From a clinic perspective a performance model only really works if all variables are known, and thus can be included in said model at the time of making a decision. In this case that decision would be made about an hour after finishing the operation when the patient has stabilised in the PACU. Looking at your Table 1 model - the PACU period variables may not be known then. I assume the period for collecting these variables were from entering PACU to leaving it. Unfortunately, if I want to have a model assist me with my decision to transfer a patient to ICU post-operatively I will not know the total time in PACU for example, I will only know how long they have spent so far. Based on this I think including pre-operative and intra-operative variables only is fair. Otherwise your model will be including information that the person using the model will not have access to at the time of making their decision.

*Author response: We agree on the point the reviewer raises here, we envisioned the prediction model to be used as decision support tool at discharge from the PACU. At the time of discharge, the time in PACU will be known and can then be included in the prediction and support the final decision on discharge. We agree with the reviewer that some PACU period variables may only be available at the end of the PACU stay, and that inclusion of pre-operative and intra-operative variables may be fair for use in prediction tools. And the reviewer is right that preoperative and intra-operative variables predict unanticipated icu admission, however our data seem to indicate that the model improves when PACU variables are included in the model.*

Figure 1
- Please tidy up the arrows so they make contact with boxes. Suggest making this in Powerpoint (much easier for arrow aligning). Also make it a consistent colour (all blue border or all black border).

*Author response: A revised Figure 1, after revision of the manuscript Figure 2, was inserted in the manuscript.*

In the exclusion group a few queries:
- What do you mean by "delayed ICU records"

*Author response: During manual review of the EMR we could read that the patient was transferred from the PACU to ICU directly but the first record in ICU was registered in the EMR longer than the two hours we used as cut-off.

The delayed ICU records note was marked with a ^*^ and the footnote of Figure was extended with explanation.*

- Why did you exclude "small surgeries"? Did you then include their second surgery as the index operation?

*Author response: Yes, their second main surgery would become the index operation. Each surgery in the study period had a separate record in our dataset, therefore the records from small surgeries such as placement of double J-stents could be excluded.*

*The small surgeries note was marked with a ^#^ and the footnote of Figure was extended with explanation.*

- "Surgeries related to previous surgeries" - what does this mean? Was this their second surgery that you were meant to exclude?

*Author response: For example, closure of an abdominal open wound treatment after a large abdominal surgery previously. Or removal of a hip prothesis because of infection after placement previously.*

*This note was also marked with a ^&^ and footnote adjusted.*

Table 1
- For continuous variable stipulate your units increase. I assume it is +1 year for age and +1kg/m^2 for BMI etc but this needs to be clarified, particularly for time in operating theatre etc.

*Author response: The reviewer’s assumptions are correct. The footnote of Table 1 was extended by the following: For continuous variables the units of increase are the following: 1 year for age, 1kg/m^2^ for BMI, 1 point for ASA score, 1 beat per minute for heart rate, 1 minute for time in operating theatre and 1% for oxygen saturation.*

- ASA score is not continuous, it is a categorical variable. I would suggest coding it as such.

*Author response: ASA is actually an ordinal scale which means that with increasing ASA an increased risk score is assigned. Therefore, it was discussed in our team that treating it as numerical is more appropriate, as an ordinal scale is usually treated as a numerical variable.*

- For the continuous variables did your analysis fulfill the linearity assumption? Clarifying this is important. If these did not fulfill that assumption they need to be altered to categorical variables, or adjusted accordingly (log adjust etc)

*Author response: No statistical tests were used to determine linearity. However, variables were visually inspected by taking a look at plots of each predictor versus the logit predictions. Possibly explained by the small number of cases, only linear relationships were found.*

*This clarification was added to the manuscript in the Methods section in Line 157-158: All continuous variables were plotted against the logit predictions and visually inspected to determine linearity.*

L216 How did they determine the complications arose from infectious causes?

*Author response: An infectious cause was determined based on a combination of clinical parameters, increased inflammatory lab results and sometimes positive cultures for which antibiotics were started.*

*This sentence was added in the footnote of the S4 Table.*

Discussion:
- Overall well written and clear. I do challenge the authors to explain where, how and why this model will not be used. Many papers produce prediction models of varying quality, but very few explain how they will use this data. As a reader I can't use this information to implement in my own center, are you now using it at your own hospital? Is it integrated in your EMR now? It is important to not only produce the prediction model, but to also implement it. This paper provides an example of what to be careful of (<https://www.bmj.com/content/369/bmj.m1328>). Published Jan 2021 found 232 prediction models centered around COVID, but only 2 models validated in multiple cohorts that may be appropriate for implementation.

*Author response: The mentioned paper is highly interesting and relevant to our research. We will indeed aim for clinical implementation, but further validation of the model is warranted.*

*As explained before, our wish is to eventually develop a tool which is implemented in our new EMR and uses real-time information to automatically calculate a risk probability score for later unanticipated ICU admission which helps the anesthesiologist at the end of PACU stay to decide whether the patient should be admitted to ICU. In addition, the majority of hospitals in the Netherlands is supported by the same type of EMR. We will test the model in a multicenter study, but first validation in our own hospital in the new EMR is required.*

*We realize that some extra focus could be laid upon the limitations and future applicability. Therefore, the Discussion section was extended with the following sentence in Line 314-316: The next step is the development of a digital tool in the EMR to automatically assign risk scores and to validate this tool in multiple centers.*

Reviewer #2: The investigators undertook a retrospective review of electronic patient information in a hypothesis-generating attempt to better understand factors that influence admission to the intensive care unit after an operation. They are particularly interested in what they define as an unplanned admission.

I congratulate the team for examining factors that may help to inform their clinical team at their institution. This seems like the beginning of a quality improvement project (a needs assessment). As I understand this, the investigators are trying to identify factors that may be missed from clinicians who plan admissions to the ICU. Or factors that arise during the operation. Other reports in the literature do not focus on unplanned admissions because this outcome is not all that well defined and other more meaningful outcomes (morbidity and mortality) are typically studied in this context.

*Author response: Thank you for the compliments and review of our manuscript. We hope to answer your valuable questions point by point.*

However, I could see how the authors' institution would be interested in the unplanned admissions for planning and cost purposes. Ultimately in the clinical setting, the most interesting outcome is failure to rescue and this is a minor component of that outcome. I would like to better understand the implications of this research - for example, was the cost different for planned/unplanned or were the patient outcomes (morbidity/mortality) worse? That analysis could help to understand why this investigation is important.

*Author response: The implications of this research are less unanticipated ICU admissions and consequently, less costs in prolonged length of stay in the hospital. In addition, management of capacity in ICU beds or operating theatre spaces could be optimized. The ultimate goal would be to decrease morbidity and mortality but the study is not powered to provide an answer.*

Are there times where patients have to recover in the PACU because ICU bed space is not yet available? If so, how do you account for this because these patients would not actually be unplanned? If not, does that mean that your ICUs are underutilized?

*Author response: In our hospital, the situation where patients recover in PACU because an ICU bed is not yet available hardly ever occurs. It was estimated to be extremely rare and did not occur in any of our cases, therefore we did not account for this situation in our analysis.*

The authors acknowledge the limitations of not having a testing and validation cohort. This does seem like a rather substantial limitation to any conclusions that could be drawn. There is not likely anything that can be done at this point, but it is a concern.

The authors also acknowledge the challenges of picking variables. I agree that this might be the biggest limitation to their analysis. I may not be understanding the analysis correctly, but wouldn't some of these variables be potential mediators for unplanned ICU admission. For example, PACU time is predictive of unplanned ICU admission, but wouldn't there be reasons that patients are spending more time in recovery and then to help clear recovery patients would be transferred to the ICU instead of the ward?

*Author response: The reviewer is partly pointing out the same concerns as reviewer #1 and we would like to refer to our answer on page 2 and 3 of this document to explain why we included PACU time for example.*

*Regarding the last part of the comment, we could not think of instances where recovery patients would be transferred to ICU because no bed is available at the ward. In our hospital, a patient is only going for surgery when an postoperative hospital bed is available.*

Did the authors investigate collinearity with the inclusion of so many variables?

*Author response: Collinearity was not investigated specifically since our main goal was prediction. However, a forward/backward variable selection was used to reduce collinearity. The forward/backward selection will on each step only add the variable with the highest prediction gain to the model . This prevents two highly correlated variables from being added since the prediction gain from the second variable being added would be very low.*

Did the authors consider the potential use of a mixed model for healthcare providers (anesthesiologist or surgeon who did or did not plan ICU admission) to account for random effects?

*Author response: Thank you for suggesting this interesting research question, but in our hospital, the decision for ICU directly from PACU is made by the anesthesiologist, and the decision for unanticipated ICU admission is made by the intensivist. This decision is based on the type of surgery, comorbidities and other rather fixed protocols based on vital parameters. Because there are no mixed roles concerning the admission of patients to ICU and no expected differences between clinicians whether to admit a patient to ICU, we did not correct for this effect.*

It appears that the investigators used BMI as a continuous variable. The extremes are often the most interesting. I would be curious about a categorical variable as malnourished or underweight individuals (BMI<18) might behave differently. There are no other markers of malnutrition in their model.

*Author response: This would certainly be a possibility but the BMI was kept as a continuous variable since this has a higher fidelity compared to a categorical.*

With this data, it would be interesting to examine mortality after unplanned ICU admission. This description and better understanding of patients who experience failure to rescue would be even more clinically important.

*Author response: This reviewer’s suggestion was taken into account and the mortality rates were added to the Result’s section in Line 184. In such a small group, it will be hard to draw valid conclusions for subgroup of mortality after unanticipated ICU admission.*

The authors note the differences in their setting with high-resource areas like the United States, but it would be helpful for readers to also understand the context with low-resource settings. Given that the ICU bed space is even more limited in other areas of the world (<https://doi.org/10.1016/S2214-109X(21)00291-6)>, the authors could comment on the utility of prediction in these settings particularly with interest in failure to rescue after surgery (DOI: 10.1097/sla.0000000000005215 and <https://doi.org/10.1111/anae.14934>).

Author response: The reviewer points out that the other side of the spectrum could be discussed as well. The proposed reference was interesting to include in our Discussion section in Line 300-301, following the added sentence: “And on the other end of the spectrum, an estimated five billion individuals in low-resource countries are subject to delays and shortages in perioperative care”.

Reviewer #3: In their interesting manuscript, the authors describe the course of patients with not anticipated secondary ICU admission in the period 2013-2017. They describe factors contributing to the need for ICU care and the outcome of this patient population. In general the manuscript is acceptable written and require some language editing, the topic is of interest and clinical relevance for the community.
There are some topics which require attention and most likely a revised version of the manuscript.

*Author response: Thank you for your review and suggestions to improve our manuscript. The suggestions and questions were addressed point by point below.*

1. The authors present data from 2013-2017, as we know the population of patients has been changing in the last years due to shared decision making and a change in surgical approach. Do the authors think that the population is still valid in 2022?

*Author response: We think the population is still valid as no large changes were made in medication prescription use, type of anesthesia of surgical techniques nor ICU admission criteria. The only change was a change in EMR, therefore data was collected until 2017.*

*This was explained in the Methods section in Line 127 that no large changes took place in this period.*

2. In table 4 the authors gave an overview about the underlying reason for ICU admission. I was wondering why sepis/sirs etc. was placed in the field cardiovascular= in particular as vasopleagia was placed in a different entity, please comment?

*Author response: Thank you for pointing out this unclear description. Vasoplegia was placed in this category because epidural anesthesia sometimes induces a vasoplegic state for which vasopressors are needed. Table S4 was adjusted to explain this point better.*

3. The authors used phenylehrine boluses and continuous vasopressor infusion as parameters, I think the use of vasopressors should be stated instead of phenylephrine.

*Author response: The word phenylephrine was changed into vasopressors throughout the manuscript.*

4. The in-hospital mortality of the ICU group was 13.9%, it will be interesting if the patients died in the ICU or in the hospital, please add. (Table 2)

*Author response: This was added in the Results section in Line 191.*

5. It is more common to describe it as Anesthesia technique instead of Method of Anesthesia, probably an exchange is good.

*Author response: This suggestion was changed accordingly.*

6. One of the patient populations at risk is the group of patients receiving betablockade for secondary prophylaxis. Is the use of an increased heart rate really justified. Do the authors used other drugs than anti-coagulation drugs as part of the risk analysis and was the prescription behavior consistent in the timeframe from 2013.-2017.

*Author response: The reviewer points out one of the limitations of using heart rate as a variable as it is susceptible to many confounders, such as betablockade use, pain, stress etcetera. Regarding prescriptions of medication, it was not possible to extract exact information in the EMR in terms of dosage and exact prescription on the day of surgery. Because it is still likely to be relevant for predictions even when some confounding could be going on, we choose to include heart rate to analyze the predictive value.*

7. The group of general surgical patients contributed significantly two questions:
a. Are vascular surgical patients part of this group?

*Author response: Indeed, vascular surgery was included. This was added in Line 140.*

b. can you split up in cancer non-cancer surgery or do you think this is not necessary?

*Author response: Large abdominal cancer surgery already has anticipated ICU admission because of increased risk of anesthesia. For the remaining surgeries, the risks related to cancer are also taken into account in the ASA Physical Status classification.*

8. For me cerebral infarction is rather cardiovascular then heamatological and major bleeding in my opinion does also not fit within that group, do you think it may be useful to re-group parts of this table? Have you thought about working with MACE like endpoints?

*Author response: At the time of designing that table, literature was searched for a general classification. We found it challenging to find a uniformly used classification and therefore used the current classification by type of organ dysfunction. The MACE endpoints do cover a number of causes but seem more appropriate for specific types of surgery. Thanks to the reviewer we realize that there is no added value to try to classify the causes. We propose to remove the first column of the table and just list the causes of unanticipated ICU admission.*

9. In the meantime, there are much better references for Bayesian network analysis than a textbook (probably some examples from the CoVid literature)

*Author response: This suggestion will improve the manuscript, the reference was changed into “Bayesian Networks for Risk Prediction Using Real-World Data: A Tool for Precision Medicine” from ScienceDirect.*

10. I found the number of patients with a saturation below 85% quite impressive, any references about this topic showing that this is in line with the benchmark in the literature?

*Author response: A comparable study by Petersen Tym et al reports 7.2% desaturation below 90% in 7.2% of the patients during their PACU stay. This was prospectively collected and the manuscript did not describe how it was measured or registered. In our retrospective study, any measurement of pulse oximetry with saturation below 85% was registered. The desaturation could be a very short moment, therefor we could explain the numbers in our study. We included it into our dataset instead of excluding it as an outlier, as we intended to analyze the significance of these short moments of desaturation in relation to predictive value.*

11. The use of the word `more` seems not always appropriate, please do some language spelling,

*Author response: Thank you for pointing out this repeatedly used word. The manuscript was checked and changed where appropriate:*

*Line 83: “more” removed.*

*Line 94: “more” removed.*

*In Line 214: “more” was replaced by “have a stronger correlation”.*

*In Line 265: “more” removed.*

*In Line 321: “more” was replaced by “have greater accuracy”.*

12. The argumentation that data is used in a period with an unchanged EMR is quite relevant.

*Author response: we agree that this is relevant, therefore it was mentioned in Line 129.*

12 For my interest, why do you use MATLAB AND SPSS programming for the forward and backward analysis, is there reasoning?

*Author response: SPSS was used for the univariate analysis. However the multivariate model was constructed in MATLAB since the investigator who perfomed the final analysis was more experienced in MATLAB.*

*Since the same results in univariate analysis were established with MATLAB compared to SPSS, the information that both systems were used seems irrelevant. For clarification reasons, we removed SPSS in Line 154-155 and 166.*

Reviewer #4: In this study, the investigators used EMR data to predict unplanned ICU admissions. Prevalence of this outcome was rare, occurring in <1% of patients. This was a single center study with a fairly large sample size overall, although the authors did not provide how many patients were excluded due to missing covariates in the multiple regression model. The most important finding was that both peri- and pre-operative data contribute to improved prediction of ICU use. I have some concerns about the robustness of the performance evaluation presented. Also, justification for such a model as a decision support tool was lacking. Improving these areas of the paper would be necessary to justify publication. Specific comments are below.

*Author response: Thank you for your thorough review and valuable suggestions. We hope that the answers to your questions meet the expectations.*

• What decisions are affected by this model? It is described as a decision support tool, but no decisions are described. It is unclear when and how the model should be used.

*Author response: thank you for pointing out, consistent with other reviewers, that we could improve our intentions. The intended use of this model would be to implement it as a prediction tool into the EMR and automatically assign a patient who reaches the end of his PACU stay a low / medium/ high risk for unanticipated ICU admission later on. The reason we did not report on this tool is because we did not do the analysis yet and have not yet validated the tool.*

• The STROBE diagram should include missing data and then the final boxes should be the dataset analyzed after excluding the missing patients

*Author response: Based on the suggestion of another reviewer, the TRIPOD diagram should be used and is now completed.*

*The missing boxes in the flowchart were added and the final groups included for analysis were mentioned in the Results section in Line 181-182: Due to missing variables, a total of 21526 controls and 179 cases were included for analysis.*

• AUC is not a robust measure for evaluating a prediction model when prevalence is very low – ICU admission rate in this study was <1%. Precision, recall, F1 should also be reported.

*Author response: Thank you for the suggestion and opportunity to make our manuscript more complete. The requested numbers were calculated and added to the manuscript in the Results section in Line 227-228 the following: The model has a calculated accuracy of 0.98, precision of 0.14 and recall of 0.17 with a resulting F1 score of 0.16.*

*The high accuracy was expected with an unbalanced dataset, which explains the low precision, recall and F1 score. These values were not published in comparable studies. Since our goal is to develop a tool for risk scores, it is known that 100% perfect prediction beforehand will be unlikely. The model/tool should be able to appoint the high risk patients and it will be accepted that a number of patient will falsely be assigned a high risk.*

Calibration and discrimination should also be evaluated.

*Author response: The calibration and discrimination was evaluated with a reliability plot, showing reasonable calibration as the plotted line is roughly through the midline.*

• Treatment of the confounder described in lines 195-198 is vague and requires clarification

*Author response: This was rephrased in the revised manuscript in Line 214-215 as: “The multivariable analysis showed that the anesthesiologists’ review had a stronger correlation with the ASA Physical Status Classification System score and duration of stay in the PACU than with unanticipated ICU admission and was therefore regarded as a confounder.”*

• Treatment of the continuous predictors as linear in the multivariable model should be justified. Did you first explore the relationships using cubic smoothing splines or lowess to determine if the relationships were approximately linear? ASA is more often treated as categorical, so justification for treating it as numerical/linear would be beneficial.

*Author response: Exploration of the variables was performed but maybe due to the small number of cases only linear relationships were found by visual inspection. ASA is actually an ordinal scale which means that with increasing ASA an increased risk score is assigned. Therefore, our team discussed to treat ASA as numerical is appropriate, as an ordinal scale is usually treated as a numerical variable.*

• Units of continuous predictors are small and result in very small odds ratios. I would consider presenting the odds ratios by 10 unit increases.

*Author response: All variables were standardized to create a fair comparison between all the variables. Comparing the odd ratios would be biased if they were based on the units.*

• Was collinearity explored? Presumably, surgery lowest HR and PACU lowest HR would be correlated. It would be preferable to present the most important predictors that don’t explain the same variability in Y.

*Author response: This point was raised by another reviewer as well. We would like to refer to our answer on page 9.*
