# Supplementary material for: Prediction of postoperative patient deterioration and unanticipated intensive care unit admission using perioperative factors
Source: PLoS One. 2023 Aug 3;18(8):e0286818. doi: 10.1371/journal.pone.0286818 (PMC10399824; doi:10.1371/journal.pone.0286818)
Supplement: Attachment — Submitted filename: Response to reviewers revision 2.docx [file pone.0286818.s011.docx]

**Response to reviewers**

Reviewer #1: I thank the reviewers for their changes and considering my comments, it is much appreciated.

Overall:

4 main problems (referring to section in my previous comments):
#1
Thank-you for the responses to my suggestions. It is now clear you are intending to build a model to establish risk of an unanticipated ICU admission that could be built into your EMR and automatically calculated and presented to clinicians. Thank you for only including the full model in the main paper, this makes the results much simpler to read.

#2
Thank-you for the response to my suggestions. This use of this variables is now much more clear.

#3
Thanky-you for the response to my suggestion. I recognise the limited number of patients with the rare outcome of unanticipated ICU admission which precludes using a large number of inclusion criterion. The use of the figure and median/IQR is very helpful and confirms that most admissions are occuring in the first few post-operative days. I

#4
Thank-you for the response to my suggestion. The intended use of the model is very reasonable and would definitely assist clinicians with deciding whether to refer their patient to ICU early in their stay to avoid unanticipated admissions post ward transfer.

Abstract:
Nil suggestions.

Introduction:
Nil suggestions.

*Author response: Thank you very much for this positive response!*

Methods:
Line 124: suggest defining the HR acronym after heart rate so it is clear you are referring to a heart rate rule in Line 127.
 *Author response: Thank you, that is a good idea.*

Discussions:
Nil suggestions.

Results:
Nil suggestions.

Figures:
Nil suggestions.

Tables:
Nil suggestions.

Reviewer #2: Thank you for addressing the questions raised. Seemingly due to study design, there remain a number of issues that are limitations to the practical implementation of this model.

*Author response: Thank you for your time. We hope the limitations were addressed according to your expectations.*

Reviewer #4: In this manuscript the authors present a multivariable logistic regression model for prediction of unplanned ICU admission after surgery. They use pre, peri, and post-operative data. This study was conducted in a relatively small data set from one institution. I have a number of concerns about the statistical methods, results, and bias in the data source that are outlined below. Because this is not novel work, some recent work in this area has not been cited, and the results are not generalizable, the significance is low.

• Data should be split into training and test sets, according to TRIPOD guidelines

*Author response: The reviewer points out one of the challenges in this study. We would like to thank the reviewer for the opportunity to improve our manuscript by implementing stratified bootstrapping to create, train and test the data. Instead, we implemented stratified bootstrapping to create train, test splits. In this way, we keep the size of the training dataset the same as the original and the number of patients in the outcome group stays the same.*

• Model selection based on p-values (i.e., stepwise, forward, backward) is outdated, especially for rather small data samples. Penalized regression techniques are preferred for a large number of covariates in relation to the number of events. False discovery rate can be added as an additional technique for variable selection after penalized regression.

*Author response: Thank you for suggesting to perform penalized regression analysis. The results were added to the manuscript. In short, the penalized regression showed comparable predictors, AUROC and AUPRC compared to our previous analysis.*

*The sections Methods, Results and Discussion were modified according to this new analysis.*

• Would the early post-operative data introduce confounding by indication?

*Author response: the early postoperative date includes only data from the PACU period. This implies that confounding by indication should be minimized as that sort of confounding is mostly induced when the patient is in the ward.*

Wouldn’t confounding by indication be present when comparing ICU admission and mortality? At the very least, there appear to be a number of mediators in the data, and this is not addressed in the statistical approach.

*Author response: The reviewer points out the duality of presenting mortality data. In the first revision round, it was requested to insert mortality data.*

*This reviewer’s suggestion was taken into account and the mortality rates were added to the Result’s section in Line 184. In such a small group, it will be hard to draw valid conclusions for subgroup of mortality after unanticipated ICU admission.*
*The ultimate goal would be to decrease morbidity and mortality but the study is not powered to provide an answer.*

• Data are referred to as singular, but they should be referred to as plural

*Author response: Thank you for notifying this inconsistency. We checked every sentence with ‘data’ and changed singular into plural throughout the manuscript. This adjusting applied to Line 172 and 245.*

• There would have been significant potential for multicollinearity of the predictors, but there is no mention of evaluation of this. Was this evaluated?

*Author response: We agree with the reviewer that multicollinearity might play a role between the different predictors. We did not report on the evaluation of multicollinearity because it is important to consider when studying causality and because multicollinearity influences p values and confidence intervals but does not influence prediction. The evaluation was performed in SPSS using correlation matrix, which showed correlation values between 0.001 to 0.334 with the majority below 0.100, suggesting low correlations. The magnitude of the standard error for each variable was inspected and within acceptable ranges. If the reviewer prefers, the results of these analysis can be added as supplemental material.*

• Inspection of the transfusion of thrombocytes in the multivariable models is necessary. Why is the CI so large? Presumably this is because it was a very rare exposure, but it may also be highly correlated with another predictor. This may cause variance inflation.

*Author response: The reviewer is right in the assumption that the transfusion of thrombocytes was very rare. In the multicollinearity analysis, there was no correlation with, for example, erythrocytes, suggesting major bleeding complication. After the implementation of penalized regression analysis instead of forward-backward selection analysis, the thrombocytes were no longer important for prediction.*

• Other performance statistics should be shown. Examples include AUCPR, discrimination plot, calibration plot. This is especially important for rare events, because AUC will be inflated.

*Author response: The AUPRC plot was added to the manuscript as Fig 4. according to the reviewer’s relevant suggestion.*

• This small sample of patients and issues with bias in the data makes the results ungeneralizable.

*Author response: We agree with the reviewer that the small sample is a limitation. However, the analyzed dataset is larger than comparable studies and can still:*

1) *serve as an example of reproducibility of previous studies*
2) *provide base for local prediction models*
3) *show the importance of including data from different phases in and around surgery that is readily available and easily found in the EMR*

• More ideas around how this model could be implemented in practice would strengthen the discussion

*Author response: In the previous revision round, the manuscript was modified to provide more ideas about implementation in clinical practice. Based on this reviewer’s point, we added the following to the Discussion section in Line 310-312: “This digital tool could automatically calculate a low, intermediate, or high risk of unanticipated ICU admission and provide decision support to the anesthesiologist in PACU.”*

• Inclusion of ROC curves does not add value to the paper.

*Author response: In medical journals, the ROC curve is frequently used, requested and interpreted, even though it might not be perfect from a statistical point of view. Therefore, we prefer to leave it in the manuscript. In addition, in the previous revision round, the extra two ROC curves were removed from the manuscript and includes as supplemental material.*

• Many of the labels for variables in the S3 table do not match the labels in Table 1, so it’s difficult to compare univariable and multivariable ORs.

*Author response: The labels were matched more clearly.*

• Some recent papers published on predictors of ICU admission after surgery are missing.

*Author response: Most papers examine factors from a different point of view, namely once the patient has stayed on the ward. Possibly, the manuscript is not clear enough in the perspective of this study where we were searching for prediction of unanticipated ICU admission from the point of view of an anesthesiologist in post anesthesia care unit. A literature search was performed once more but did not result in recent papers from this PACU perspective. We are wondering if the reviewer could mention the papers the reviewer had in mind?*
