# Supplementary material for: Prediction of postoperative patient deterioration and unanticipated intensive care unit admission using perioperative factors
Source: PLoS One. 2023 Aug 3;18(8):e0286818. doi: 10.1371/journal.pone.0286818 (PMC10399824; doi:10.1371/journal.pone.0286818)
Supplement: Attachment — Submitted filename: Response to reviewers - revision 3.docx [file pone.0286818.s012.docx]

Additional Editor Comments:

Please carefully address comments from reviewer 2, discussing limitation of this study in the Discussion section. Please, also address comments from reviewer 5.

[Note: HTML markup is below. Please do not edit.]

Reviewers' comments:

Reviewer's Responses to Questions

**Comments to the Author**

1. If the authors have adequately addressed your comments raised in a previous round of review and you feel that this manuscript is now acceptable for publication, you may indicate that here to bypass the “Comments to the Author” section, enter your conflict of interest statement in the “Confidential to Editor” section, and submit your "Accept" recommendation.

Reviewer #1: All comments have been addressed

Reviewer #2: (No Response)

Reviewer #5: (No Response)

2. Is the manuscript technically sound, and do the data support the conclusions?

The manuscript must describe a technically sound piece of scientific research with data that supports the conclusions. Experiments must have been conducted rigorously, with appropriate controls, replication, and sample sizes. The conclusions must be drawn appropriately based on the data presented.

Reviewer #1: (No Response)

Reviewer #2: Partly

Reviewer #5: Partly

3. Has the statistical analysis been performed appropriately and rigorously?

Reviewer #1: (No Response)

Reviewer #2: I Don't Know

Reviewer #5: Yes

4. Have the authors made all data underlying the findings in their manuscript fully available?

The [PLOS Data policy](https://eur02.safelinks.protection.outlook.com/?url=http%3A%2F%2Fwww.plosone.org%2Fstatic%2Fpolicies.action%23sharing&data=05%7C01%7C%7Ca09deb90c2b84929a23408db260c06ac%7Ccc7df24760ce4a0f9d75704cf60efc64%7C1%7C0%7C638145605324517313%7CUnknown%7CTWFpbGZsb3d8eyJWIjoiMC4wLjAwMDAiLCJQIjoiV2luMzIiLCJBTiI6Ik1haWwiLCJXVCI6Mn0%3D%7C3000%7C%7C%7C&sdata=xtTgbpbEZbCejzEfHjpu6nFsGWyLxeI%2FNjvwVFxp2qM%3D&reserved=0) requires authors to make all data underlying the findings described in their manuscript fully available without restriction, with rare exception (please refer to the Data Availability Statement in the manuscript PDF file). The data should be provided as part of the manuscript or its supporting information, or deposited to a public repository. For example, in addition to summary statistics, the data points behind means, medians and variance measures should be available. If there are restrictions on publicly sharing data—e.g. participant privacy or use of data from a third party—those must be specified.

Reviewer #1: (No Response)

Reviewer #2: Yes

Reviewer #5: No

5. Is the manuscript presented in an intelligible fashion and written in standard English?

PLOS ONE does not copyedit accepted manuscripts, so the language in submitted articles must be clear, correct, and unambiguous. Any typographical or grammatical errors should be corrected at revision, so please note any specific errors here.

Reviewer #1: (No Response)

Reviewer #2: Yes

Reviewer #5: Yes

6. Review Comments to the Author

Please use the space provided to explain your answers to the questions above. You may also include additional comments for the author, including concerns about dual publication, research ethics, or publication ethics. (Please upload your review as an attachment if it exceeds 20,000 characters)

Reviewer #1: (No Response)

Reviewer #2: As before, the study design limits the practical implementation of this model.

We agree with the reviewer's comment and have added to the discussion how we see the practical implementation of this model.

Changes: Added to the discussion at lines 274-277

...............................................................................................................................................

Reviewer #5: I thank the authors for the opportunity of reviewing this interesting manuscript. The topic is important and the study was well performed. The authors have addressed the requests by previous reviewers.
The message of the authors is clear but I have some doubts about the translation from theory to practice of the predictive model developed.

These are my suggestions for the authors:

The authors analyzed and built the model on data collected between 2013 and 2017. Some changes in daily practice may have occurred since 2017. This may limit the application of the predictive model. Authors should explain and comment on this.

I am not confident with penalized logistic regression which is not commonly used. This probably helps to better assess the readability of the manuscript for ordinary readers... I am puzzled by some problems:
(1) Why did the authors not report P-values for each variable in Table 1?

Thank you for highlighting this confusing aspect of the penalized regression. Using L1 for the penalized regression causes shrinkage of the beta values which are not correlated to the outcome. This improves the predictions of the logistic regression but also creates a bias in the beta values. Therefore, p-values for the beta values cannot be calculated

(2) Table 1 shows the "Most important predictors". I guess the authors mean that these are the significant predictors that are part of the final model. I suggest correcting the table title accordingly. In addition, the authors should also report insignificant predictors (perhaps as supplementary material). For example: what about ASA class 2?

We agree with the reviewer that the definition of ‘Most important predictors’ needs more clarification. The ’Most important predictors’ Table is based on the predictors for which the 95% confidence interval of the beta values over the bootstrapping is not equal to 0 (odds ratio = 1). Showcasing the other variables might indeed provide important information, and improve the reproducibility of the model.

Changes: Added explanation of Table 1 in lines 203-206

Changes: Added S6 Table with odds ratios for all predictors, referred to in lines 206-207

(3) Table 1: Increased BMI appears to be protective... this deserves a comment in the discussion section

Upon closer inspection, the BMI should not have been included in this Table according to the definition explained in point (2), since the confidence interval contains an odds ratio of 1. Thank you for making us aware of this mistake.

These are values in the multi-variable model. Correlation between variables could have caused this. Another possibility is that a higher BMI could provide some patients with some immunomodulation effects or provide some reserve to support the recovery from surgery.

(4) The final model is poorly reported. How should it work in daily practice?

This comment seems to agree with the comment of reviewer #2. The final model should be used as clinical decision support at the end of the PACU stay. The model can be used to highlight high-risk patients. Then it can be decided whether a patient should be transferred to the ICU, or maybe receive more monitoring (e.g. more frequent spot checks)

Changes: Added to the discussion at lines 274-277

(5) I do not understand what the authors mean with “Lowest heart rate” and “Highest heart rate”

These definitions can indeed be confusing. The lowest and highest heart rate refers to the lowest and highest heart rate attained during that period (e.g. the lowest heart attained during the surgery). These should match the definition in Table S1. Therefore, in the new version, the name has been changed to match those in Table S1.

Changes: Table 1, Changed the lowest heart rate to the minimum heart rate
